# Identification of a candidate dwarfing gene in Pallas, the first commercial barley cultivar generated through mutational breeding

**DOI:** 10.3389/fgene.2023.1213815

**Published:** 2023-07-04

**Authors:** Shakhira Zakhrabekova, Pallavi Chauhan, Christoph Dockter, Pavithra Ealumalai, Anastasiia Ivanova, Morten Egevang Jørgensen, Qiongxian Lu, Olesya Shoeva, Klaudia Werner, Mats Hansson

**Affiliations:** ^1^ Department of Biology, Lund University, Lund, Sweden; ^2^ Carlsberg Research Laboratory, Copenhagen, Denmark; ^3^ Department of Biology, Norwegian University of Science and Technology, Trondheim, Norway; ^4^ Department of Plant Genetics, Institute of Cytology and Genetics of Siberian Branch of the Russian Academy of Sciences, Novosibirsk, Russia

**Keywords:** erectoides, *Hordeum vulgare*, lodging, semidwarf, spike phenotype

## Abstract

Many induced mutants are available in barley (*Hordeum vulgare* L.). One of the largest groups of induced mutants is the Erectoides (*ert*) mutants, which is characterized by a compact and upright spike and a shortened culm. One isolated mutant, *ert-k.32*, generated by X-ray treatment and registered in 1958 under the named “Pallas”, was the first ever induced barley mutant to be released on the market. Its value was improved culm strength and enhanced lodging resistance. In this study, we aimed to identify the casual gene of the *ert-k.32* mutant by whole genome sequencing of allelic *ert-k* mutants. The suggested *Ert-k* candidate gene, HORVU.MOREX.r3.6HG0574880, is located in the centromeric region of chromosome 6H. The gene product is an alpha/beta hydrolase with a catalytic triad in the active site composed of Ser-167, His-261 and Asp-232. In comparison to proteins derived from the Arabidopsis genome, ErtK is most similar to a thioesterase with de-S-acylation activity. This suggests that ErtK catalyzes post-translational modifications by removing fatty acids that are covalently attached to cysteine residues of target proteins involved in regulation of plant architecture and important commercial traits such as culm stability and lodging resistance.

## Introduction

Barley (*Hordeum vulgare* L.) has a long history as crop plant and was domesticated about 10,000 years ago from its wild progenitor *Hordeum vulgare* ssp. *spontaneum.* This is based on archaeological findings from the Middle East, the area known as the fertile crescent (Badr et al., 2000; Mascher et al., 2016; Mascher et al., 2017). Barley is grown worldwide with a total production of around 150 million tons per annum (www.fao.org). Also barley breeding has a long history. Mutation breeding in barley was introduced shortly after the discovery that ionized irradiation could increase mutation frequencies in the fruit fly *Drosophila melanogaster* (Muller, 1927; Muller, 1928). First, the barley cultivars Gull and Danish Maja were used. However, the cultivar Bonus, which was known for its very high tillering capability and extremely high yield, soon became a favorite for mutation research (Gustafsson et al., 1971). Initially, chlorophyll mutants were used to optimize irradiation dosage and duration of irradiation. Chlorophyll mutants were useful since they have obvious visual phenotypes already at the seedling stage of the M_2_ generation as, for example, yellow and white plants (Gustafsson, 1940). Due to the lack of chlorophyll, the chlorophyll mutations were lethal and the homozygous mutants died at the seedling stage. However, it was soon discovered that also viable mutations could be obtained, which were possible to have in homozygous form. The most common group was the so-called Erectoides (*ert*) mutants, which are characterized by an erect, compact and dense spike, and a straw that is often short and stiff (Lundqvist, 1992). Crosses were performed between different *ert* mutants that grouped 225 mutants to 31 different loci (Supplemented Table 1). The *ert* mutants were isolated during a time when agricultural practices were largely changed. A major difference was the use of fertilizers that greatly increased the yield of crops as fertilizers promote plant growth. However, the culms were not strong enough to hold the heavy spikes and as a result plants fell over. Therefore, introduction of stiff and short-culm mutant alleles in the breeding material was very important (Dockter and Hansson, 2015). An increased interest for lodging resistance has put many historic mutants with sturdy and shorter culms in focus since they represent valuable resources for plant breeding. Today, marker assistant breeding is widely used in breeding programs since a large number of traits can be followed in many individual plants. In addition, marker screening can be automated with robotic systems. Our goal is to identify mutated genes and the exact genetic identity of the mutant alleles since that will make them available for marker assistant breeding.

Mutant *ert-k.32* was induced by X-ray in Bonus in 1947. This mutant, together with *ert-a.23* (X-ray in Bonus 1944) and *ert-a.28* (X-ray and FeSO_4_ in Bonus 1944), was subjected to field trials and all three mutants showed increased lodging resistance in comparison to Bonus. Mutant *ert-k.32* had superior stem stability and was accepted as a new cultivar in 1958 under the name Pallas. This was the first example of an induced barley mutant that was released as a commercial cultivar. Since then, plant breeders have used Pallas in crosses to develop other cultivars. For example, Pallas was crossed to Herta, which resulted in the cultivar Hellas (Gustafsson et al., 1971). Visir is another cultivar developed from Pallas through a cross with a “Long glume” barley landrace which improved resistance to powdery mildew (Gustafsson et al., 1971).

Today, short stem architecture in elite malting barley is often derived from a deficiency in the gibberellic acid hormone pathway with *sdw1* alleles of the gibberellin 20-oxidase gene (*HvGA20ox2*) dominating in many barley breeding programs (Dockter and Hansson, 2015; Xu et al., 2017). However, breeders are looking for other genetic possibilities to provide lodging resistance and *ert-k.32* could be an interesting alternative. In this work, we have identified a candidate gene, which we suggest is the *Ert-k* gene. We also describe the likely *ert-k.32* mutation responsible for the Pallas phenotype. The finding will facilitate the use of this allele for efficient marker-assisted breeding and testing in today’s elite germplasm. The *Ert-k* candidate gene encodes an alpha/beta-hydrolase, which has previously not been associated with changes in plant architecture.

## Materials and methods

### Plant material and growth conditions

In this study, we used 14 barley spring cultivars (Supplementary Table S2) and eight historic *ert-k* mutant lines (Supplementary Table S3) obtained from the Nordic Genetic Resource Center (NordGen), Alnarp, Sweden (www.nordgen.org). These lines and F_1_ plants obtained from allelism crosses were grown in greenhouses at the Department of Biology, Lund University, Sweden. Plants were grown in soil with article number 744704 from SW Horto (www.swhorto.se) in 5-L pots. Greenhouse conditions were set to 16-h light/8-h dark cycles and temperature 20°C during the day and 16°C during the night. All statistical data presented for phenotypic traits are based on measurement of 6–11 plants of each line.

### General DNA methods

Genomic DNA for PCR reactions was extracted from fresh leaves using the REDExtract-N-AmpTM Plant PCR Kit (Sigma-Aldrich, St. Louis, MO, USA) according to the manufacturer’s instructions. Sanger sequencing were performed by Eurofins Genomics, Germany. Used primers are shown in Supplementary Table S4.

PCR were performed by initial denaturation at 94°C/3 min, followed by 35 cycles of 94°C/45 s, 56°C–62°C/45 s and 72°C/60–90 s, with a final extension step of 72°C/10 min.

Purification of PCR products was done by using Illustra ExoProStar 1-Step (Cytiva, Marlborough, MA, USA), following manufacturer’s protocol.

Found mutations were confirmed by Sanger sequencing of at least two independent reactions.

### RNA isolation and RT-PCR

In order to analyze whether the eight *ert-k* mutants are transcription deficient for the *Ert-k* candidate gene, RT-PCR (not qRT-PCR) was performed. Plants were grown in soil and 100 mg material from a single leaf was harvested and immediately frozen in liquid nitrogen. Porcelain mortar was used to homogenize leaf material under liquid nitrogen. Total RNA was isolated using Trizol reagent (Invitrogen Carlsbad, CA, USA) according to the manufacturer’s instructions. Residual DNA was removed by DNAse I treatment (Thermo Fisher Scientific, USA). A 500 ng of total RNA was converted to single-stranded cDNA by using RevertAid First Strand cDNA synthesis kit (Thermo Fisher Scientific, USA) primed with (dT)15 in a 20 µL reaction volume (Stuart et al., 2021). Primers were designed to amplify a 1006 bp cDNA fragment. (Supplementary Table S4).

### Allelism tests

The *ert-k*.32 mutant was used as male in each cross. Mutants *ert-k.76, ert-k.93, ert-k.302, ert-k.309, ert-k.435, ert-k.459, ert-k.477* and their mother cultivars Bonus and Foma were used as female. Pollination was performed 3 days after emasculation. F_1_ plants were phenotyped and genotyped. For genotyping, specific primers were designed that could amplify either the wild type allele or the mutant allele (Supplementary Table S4). 6–11 F_1_ plants of each cross were evaluated. The statistical analyses were based on comparisons between a mutant and its mother cultivar, or comparisons between a mutant crossed to *ert-k.32* and its mother cultivar crossed to *ert-k.32*.

### Whole genome sequencing and data analysis

Genomic DNA for whole genome sequencing was extracted by a modified CTAB protocol (Doyle, 1991) as described in (Stuart et al., 2021). Genomic DNA was sent to the Earlham Institute (Norwich, UK, www.earlham.ac.uk) where DNA libraries were prepared and where DNA sequencing was performed on an Illumina HiSeq4000.150 bp paired-end reads were obtained.

BWA mem (version 0.7.17) was used to align reads to the barley MorexV3 reference genome (Li and Durbin, 2010; Mascher et al., 2021). Samtools fixmate (samtools version 1.10) was used to fill in mate coordinates. Aligned reads were further processed to remove PCR duplicates by running Samtools markdup (Li et al., 2009). To get vcf files, bcftools mpileup (bcftools version 1.17) was used with the following filtering options: q 60 -Q 30 -D (where -q is mapping quality for the alignment, -Q is minimum base quality, -D is instruction to run the BAQ algorithm not only in problematic regions, but on all reads) (Li, 2011). To identify alleles observed in a mutant sample and not found in the control (mother cultivars Bonus or Foma), bcftools in combination with the “contrast” plugin was used. Bcftools view was used to exclude sites with heterozygous genotypes (-g ^het), and to exclude sites with the reference genotype (-e ‘GT = "ref"'). Functional annotation of variants was performed with Ensembl Variant Effect Predictor (McLaren et al., 2016).

## Results

### Genomic sequencing of *ert-k* mutants to identify an *Ert-k* candidate gene

**FIGURE 1:**
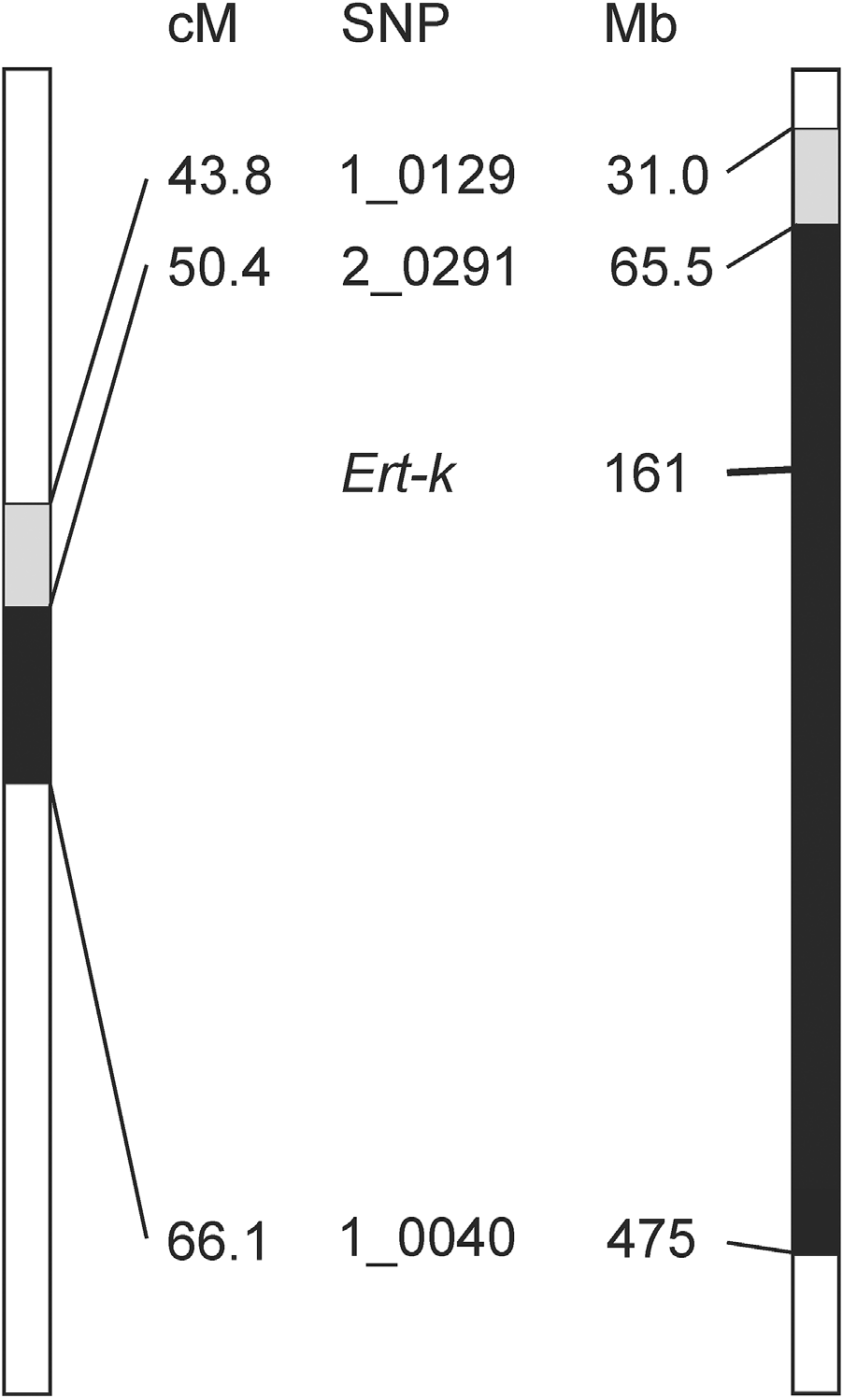
Genetic (left) and physical (right) maps of barley chromosome 6H showing the introgression region between markers 1_029 and 1_0040, linked to *ert-k.32* according to (Druka et al., 2011). The mapping was further improved by (Skov Kristensen et al., 2016) (marked in black). *Ert-k* candidate gene HORVU.MOREX.r3.6HG0574880 is located at position 161752829–161757701 Mbp in the physical map. Positions in Mbp are according to version 3 of the barley cultivar Morex (Mascher et al., 2021). Genetic position of SNPs is according to IBSC (2012) and name of SNP markers is according to (Close et al., 2009).

The *Ert-k* locus is represented by eight different alleles (Franckowiak and Lundqvist, 2012), which have been generated through treatment by X-rays, gamma-rays, neutrons and ethyl methanesulfonate of the cultivars Bonus and Foma (Supplementary Table S3). Early mapping experiments demonstrated that *Ert-k* is located on chromosome 6H (https://bgs.nordgen.org/). More recently, mutant *ert-k.32* was backcrossed to the cultivar Bowman to create a near-isogenic line; BW314. By genotyping BW314 with 3,072 single nucleotide polymorphisms markers (SNPs), the introgression region was defined by 14 SNP markers spanning 22.3 cM (Figure 1) (Druka et al., 2011).

In our previous research, we further fine mapped the *Ert-k* locus by using three mapping populations; one double-haploid population derived from BW314 (*ert-k.32*) crossed to cultivar Quench, and F_2_-mapping populations derived from crosses of BW314 to Bowman and Quench. The analysis confirmed the centromeric location of *Ert-k* on barley chromosome 6H and mapped *Ert-k* to a 15.7 cM region between markers 2_0291 and 1_0040 (Skov Kristensen et al., 2016). With help of the barley reference genome sequence (Mascher et al., 2021), it is now possible to translate the genetic map to the physical map. The distance between 2_0291 and 1_0040 is more than 400 Mbp comprising 73% of chromosome 6H (Figure 1). There are 1,986 high-confident genes between markers 2_0291 and 1_0040. Since recombination events are less frequent in centromeric regions, fine mapping is problematic. We therefore decided to search for the *Ert-k* gene by whole genome sequencing of allelic *ert-k* mutants. Among the eight *ert-k* mutants, we selected the five mutants which had been induced by X-rays, gamma rays and ethyl methanesulfonate (Supplementary Table S3). In total, severe homozygous mutations were found in seven genes on chromosome 6H of the five mutants compared to their respective mother cultivar (Table 1). These mutations are likely to be caused by the mutagenic treatment and cause frameshifts or missense mutations changing amino-acid residues in their respective protein.

**TABLE 1:** *Ert-k* candidate genes identified through genomic DNA sequencing of five *ert-k* mutant lines. The candidate genes have homozygous mutations causing severe disruptions of the corresponding proteins. The gene HORVU.MOREX.r3.6HG0574880, encoding an alpha/beta hydrolase, was mutated in three out of the five mutants.

Gene	Annotation	Location on chr 6H (bp)	Mutant/Mother cv	Mutation	Mutant
HORVU.MOREX.r3.6HG0545860	Leucine-rich repeat receptor-like protein kinase family protein	16,575,397	C/T	missense	*ert-k.32*
HORVU.MOREX.r3.6HG0565880	Polyubiquitin	96,640,536	A/G	missense	*ert-k.32*
		96,640,536	A/G	missense	*ert-k.76*
HORVU.MOREX.r3.6HG0567140	Pentatricopeptide repeat-containing protein	106,308,104	A/G	missense	*ert-k.435*
HORVU.MOREX.r3.6HG0574080	Recombination-associated protein RdgC	155,029,203	C/G	stop-gain	*ert-k.32*
HORVU.MOREX.r3.6HG0574880	Alpha/beta-Hydrolases superfamily protein	161,755,966–161,755,969	4 bp deletion	frameshift	*ert-k.32*
		161,755,966–161,755,969	4 bp deletion	frameshift	*ert-k.76*
		161,757,170	1 bp insertion	frameshift	*ert-k.309*
HORVU.MOREX.r3.6HG0575620	NAC (No Apical Meristem) domain transcriptional regulator superfamily protein	167,929,576	T/C	missense	*ert-k.32*
		167,929,576	T/C	missense	*ert-k.76*
HORVU.MOREX.r3.6HG0624670	BED zinc finger, hAT family dimerization domain	540,945,225	T/A	missense	*ert-k.477*

**FIGURE 2:**
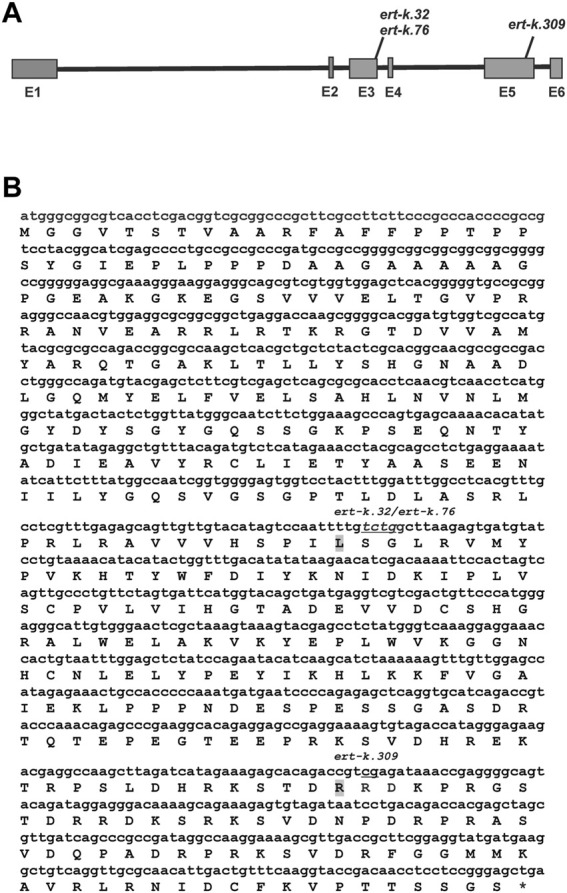
**(A)** Structure of the barley gene HORVU.MOREX.r3.6HG0574880, suggested to be *Ert-k,* and the position of detected mutations. **(B)** The cDNA and deduced polypeptide of the suggested *Ert-k* gene encoding an alpha/beta hydrolase. The 4-bp deletion of *ert-k.32* and *ert-k.76* is underlined. The mutation in *ert-k.309* is an insertion of “a” between the underlined “cg”. The last native amino-acid residue in the truncated proteins of *ert-k.32/76* and *ert-k.309* are indicated in grey.

**FIGURE 3:**
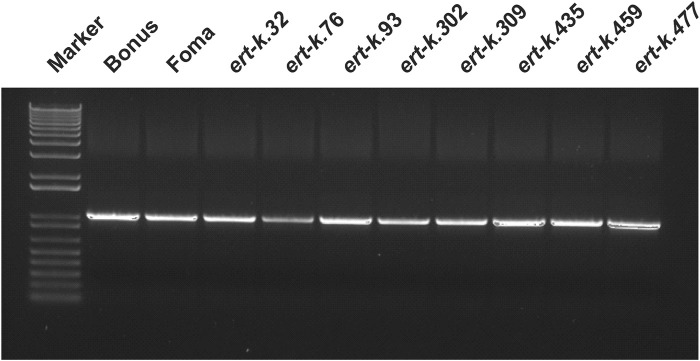
RT-PCR analysis of HORVU.MOREX.r3.6HG0574880, suggested to be *Ert-k.* The gene can be amplified in all *ert-k* mutants and their mother cultivars.

Since the *ert-k* mutants are supposed to be allelic, we especially looked for genes where all five mutants would have a mutation. Such gene was not found. However, the gene HORVU.MOREX.r3.6HG0574880 got our attention since this gene was mutated in the coding region of three of the five analyzed mutants (Table 1). The gene encodes an alpha/beta hydrolase of 399 amino-acid residues, has 6 exons and contains a fermentation-respiration switch protein FrsA domain (Figure 2). Sanger sequencing of HORVU.MOREX.r3.6HG0574880 in all eight available *ert-k* mutants revealed an identical 4-bp deletion in exon 3 in both mutant *ert-k.32* and *ert-k.76*, and a one-bp insertion in exon 5 in *ert-k.309*. In *ert-k.32* and *ert-k.76*, the 4-bp deletion causes a truncated protein of 193 native amino-acid residues followed by an alanine residue. In *ert-k.309*, the one-bp insertion in exon 5 causes a frameshift. The truncated protein has 333 native amino-acid residues followed by glutamine and arginine (Figure 2). No mutations in HORVU.MOREX.r3.6HG0574880 were found in *ert-k.93*, *ert-k.302*, *ert-k.435*, *ert-k.459* or *ert-k.477*. We therefore analyzed whether we could detect any mRNA of the candidate gene in the eight mutants. mRNA was detected by RT-PCR in all *ert-k* mutants, which suggested that the mutants are not transcript deficient (Figure 3). Since genomic DNA sequence information was available for *ert-k.435* and *ert-k.477*, we looked for mutations in the intergenic regions in the vicinity of the candidate gene. In *ert-k.477*, we found a “C/T” SNP variation at position 6H:161619756. The mutation was confirmed by Sanger sequencing. The SNP is 133,213 bp upstream from the start codon of HORVU.MOREX.r3.6HG0574880. Three genes are located between the SNP and HORVU.MOREX.r3.6HG0574880, namely, HORVU.MOREX.r3.6HG0574850, HORVU.MOREX.r3.6HG0574860 and HORVU.MOREX.r3.6HG0574870. It is known that enhancers can be located hundreds of thousands base pairs away from the target gene and do not necessarily operate on the closest promoter (Pennacchio et al., 2013).

### Diallelic crosses to identify allelic *ert-k* mutants

**FIGURE 4:**
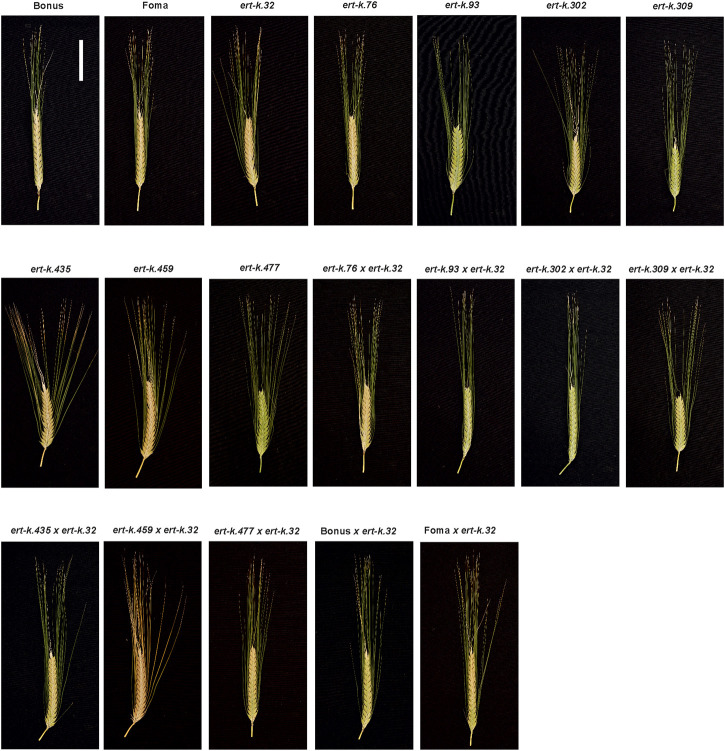
Spike phenotypes of *ert-k* mutants, their mother cultivars Bonus and Foma, and F_1_ plants from crosses. Scale bar 5 cm.

In order to analyze if the eight *ert-k* mutants are truly allelic, we crossed *ert-k.32* with the other seven *ert-k* mutants or with cultivars Bonus and Foma. Mutant *ert-k.32* was used as father in the crosses and the success of the crosses was verified by the heterozygous presence of the *ert-k.32* allele in the resulting F_1_ plants. If two recessive mutants are deficient in the same gene, i.e., truly allelic, the F_1_ generation will show the mutant phenotype. In contrast, if the two recessive mutants are deficient in two different genes, a wild-type phenotype is expected in F_1_ plants. F_1_ seeds were planted in the greenhouse together with Bonus, Foma and each of the eight *ert-k* mutants. The plants were phenotyped by visual inspection (Figure 4) and spike length and the length between rachis internodes 5 and 15 were measured (Table 2). The allelism test suggested that mutants *ert-k.93* and *ert-k.302* might be non-allelic to *ert-k.32*, which would explain why no mutations in HORVU.MOREX.r3.6HG0574880 were found in these two mutants. Also *ert-k.435* and *ert-k.459* showed no mutations in this gene although the obtained F_1_ plants displayed a mutant phenotype. Since we have no genome sequence data of *ert-k.459*, we could not analyze this mutant for intergenic SNP variation. In our previous work with barley mutants, we used to identify approximately 75% of the mutations in historic accessions that are supposed to be allelic (Zakhrabekova et al., 2012; Zakhrabekova et al., 2015; Matyszczak et al., 2020). Because the *ert-k* phenotype is similar to other *ert* phenotypes, we sequenced all *ert-k* mutants for the miRNA172 binding site of *Ert-r (APETALA2)* (Houston et al., 2013). The reason why we sequenced only the miRNA172 binding site of *Ert-r* is that it is known that only mutations within this domain are causing a dwarf phenotype (Houston et al., 2013). No mutations were found (not shown).

**TABLE 2:** Phenotypic characters of F_1_ plants obtained from crosses. The data is based on 6–11 plants of each line. The awns were not included in the spike length. The rachis internode length is the length between rachis node 5 and 15 as counted from the base of the spike. The *p*-values are based on comparisons between a mutant and its mother cultivar, or comparisons between a mutant crossed to *ert-k.32* and its mother cultivar crossed to *ert-k.32*.

	Spike			Rachis internode		
Name of the plant	Length (cm)	Standard deviation	*p*-value	Length (mm)	Standard deviation	*p*-value
*ert-k.32*	75.2	11.37	2.9 × 10^−6^	26.6	1.63	3.5 × 10^−11^
*ert-k.76*	79.7	10.15	5.5 × 10^−6^	26.8	2.07	1.3 × 10^−10^
*ert-k.93*	75.6	11.64	6.6 × 10^−6^	25.2	2.02	3.3 × 10^−11^
*ert-k.302*	82.7	8.45	0.18	26.7	1.43	4.8 × 10^−6^
*ert-k.309*	69.3	6.71	1.5 × 10^−4^	24.4	1.88	3.2 × 10^−8^
*ert-k.435*	83.6	7.8	0.3	23.1	1.34	7.9 × 10^−8^
*ert-k.459*	82.5	8.63	0.17	25.7	0.98	1 × 10^−7^
*ert-k.477*	79.9	9.22	6.3 × 10^−2^	22.1	2.01	1,6 × 10^−9^
Bonus	105.5	12.46		35.2	1.85	
Foma	89.6	12.40		31.5	1.74	
*ert-k.76 x ert-k.32*	86	10.18	1.9 × 10^−3^	26.0	1.23	4.4 × 10^−8^
*ert-k.93 x ert-k.32*	91.6	9.72	1.1 × 10^−2^	33.0	2.38	0.4
*ert-k.302 x ert-k.32*	104.4	8.71	0.38	30.8	0.85	1 × 10^−4^
*ert-k309 x ert-k.32*	78.8	9.67	3.8 × 10^−5^	25.2	1.49	4.2 × 10^−8^
*ert-k.435 x ert-k.32*	96.5	7.78	3.9 × 10^−2^	29.5	1.51	1 × 10^−4^
*ert-k.459 x ert-k.32*	88.2	5.83	7.3 × 10^−5^	29.7	1.91	7 × 10^−5^
*ert-k.477 x ert-k.32*	89.8	7.06	1.3 × 10^−3^	26.7	0.94	3.1 × 10^−7^
Bonus *x ert-k.32*	104.3	9.05		32.3	1.12	
Foma *x ert-k.32*	108.9	10.2		33.6	1.60	

### Genotyping suggests that *ert-k.32* and *ert-k.76* have different origin

**FIGURE 5:**
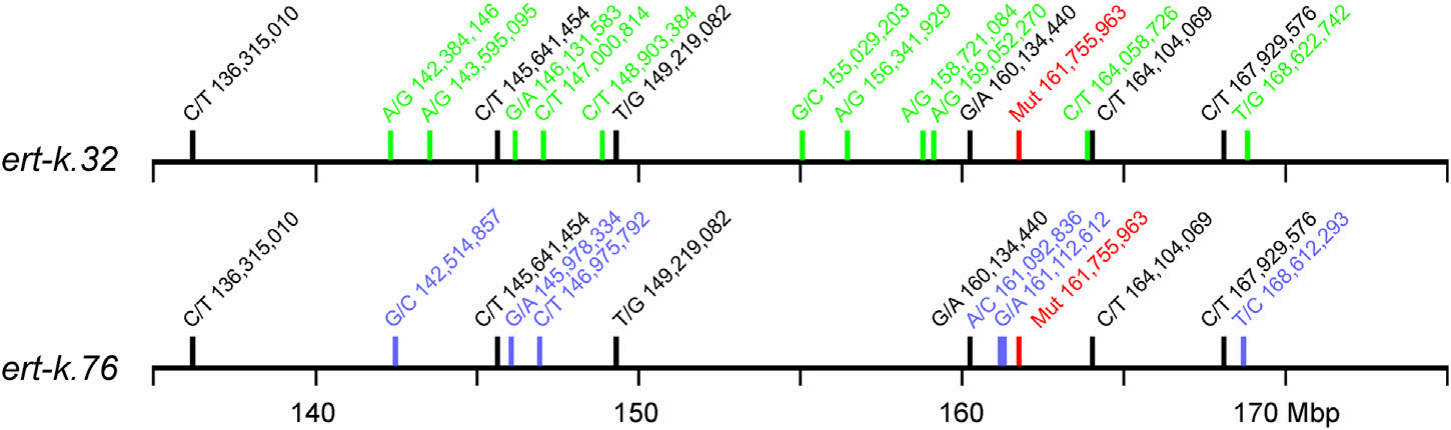
Location of homozygous SNPs in mutants *ert-k.32* and *ert-k.76* on chromosome 6H between Mbp 135 and 175. Black, SNPs common to *ert-k.32* and *ert-k.76*; green, SNPs unique to *ert-k.32*; blue, SNPs unique to *ert-k.76*; red, the location of the 4-bp mutation at bp 161,755,966–161,755,969.

Mutants *ert-k.32* and *ert-k.76* were found to have the same 4-bp deletion, which was unexpected since the mutagenic process is random and they were induced with different mutagens and were isolated in different years (Supplementary Table S3). To rule out whether the two mutant lines have been mixed up over the years or if they represent two identical mutations generated independently of each other, we analyzed the SNP variation in a 40 Mbp region around the suggested *Ert-k* candidate gene HORVU.MOREX.r3.6HG0574880. Due to the tight linkage in the centromeric region around this gene, it is very likely that existing homozygous SNPs are inherited together. The presence of identical SNPs around the mutation would indicate a common origin of the mutants, i.e., seeds had been mixed up over the years. In contrast, the presence of unique SNPs would indicate that the two mutants are the results of two independent mutagenic events. In *ert-k.32* and *ert-k.76* we identified 17 and 12 SNPs, respectively, within the 40 Mbp region. Six of these were present in both mutants, whereas the remaining 11 and 6 SNPs were unique to each mutant (Figure 5). The six common SNPs probably reflect differences between the Bonus line used for mutagenesis in 1947 and 1955 that generated *ert-k.32* and *ert-k.76*, respectively, and the Bonus accession we sequenced in this experiment. The unique SNPs were likely obtained through the mutagenic treatment. In order to investigate whether the 4-bp deletion was a result of the mutagenic treatment or was already present in an “ancient” Bonus line, we sequenced the site of the 4-bp deletion from all Bonus accessions available at the Nordic Genetic Stock Center (www.nordgen.org) and the cultivars Gull, Opal Abed, Maja Abed, Binder Abed, Guld Svalöf, Seger and Hanna, which are in the pedigree of Bonus. None of the listed cultivars showed the 4-bp deletion, which supports that the deletion is a result of the mutagenic treatment.

### Analysis of Pallas accessions and Pallas derived cultivars

**FIGURE 6:**
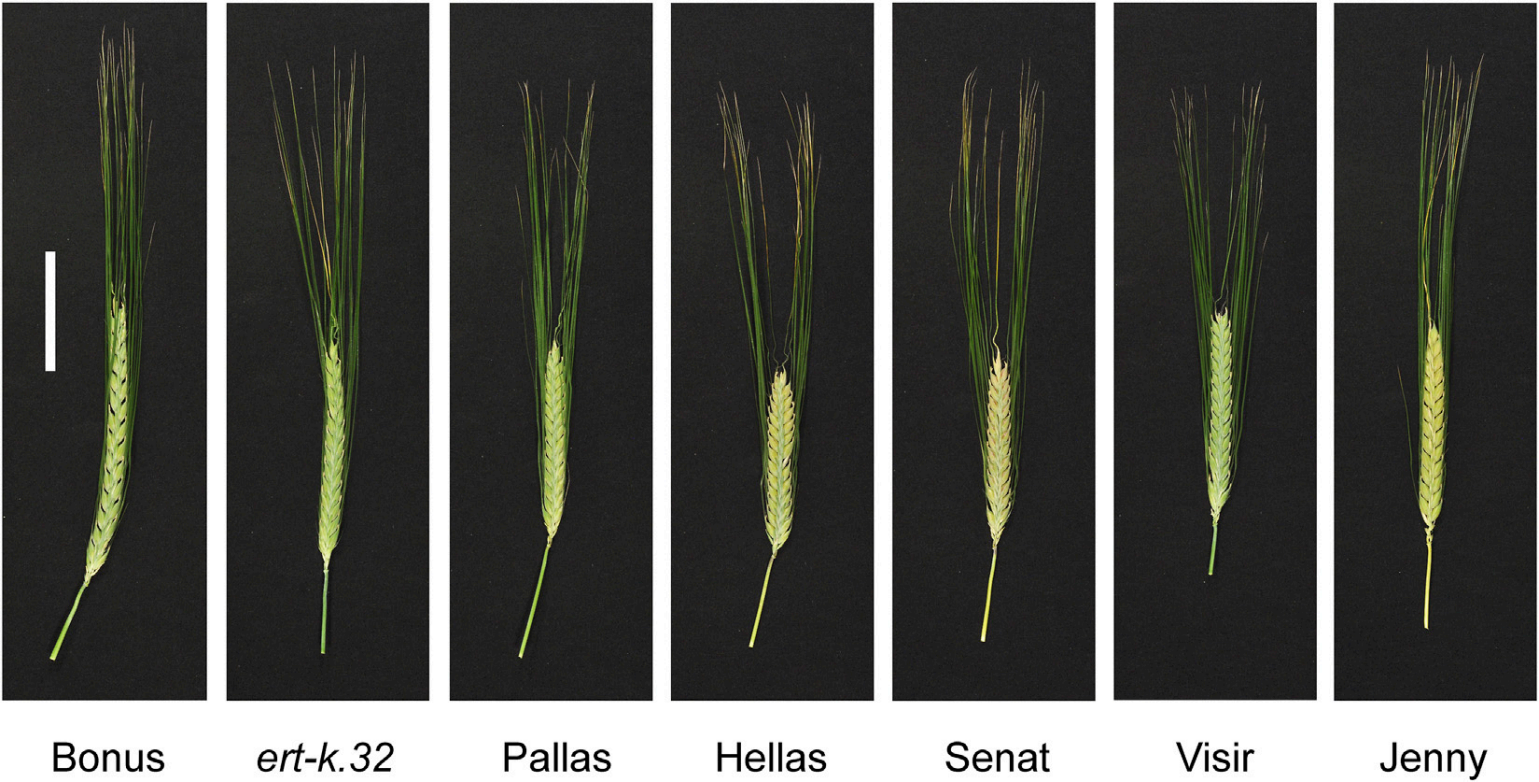
Spike phenotype of cultivars with Pallas in their pedigree. Scale bar 5 cm.

Since Pallas was used in plant breeding shortly after it was released as a cultivar, we analyzed cultivars with Pallas in their pedigree and four accessions of Pallas that are available at the Nordic Genetic Stock Center (www.nordgen.org) (Supplementary Table S5). The 4-bp deletion seen in *ert-k*.32 and *ert-k*.76 was found in all cultivars except Jenny, which in addition did not show the characteristic compact spike phenotype of Pallas (Figure 6). The fact that all four Pallas accessions and the cultivars Hellas, Senat and Visir contained the 4-bp deletion further supports that HORVU.MOREX.r3.6HG0574880 is the *Ert-k* gene.

### Identification of a catalytic triad

**FIGURE 7:**
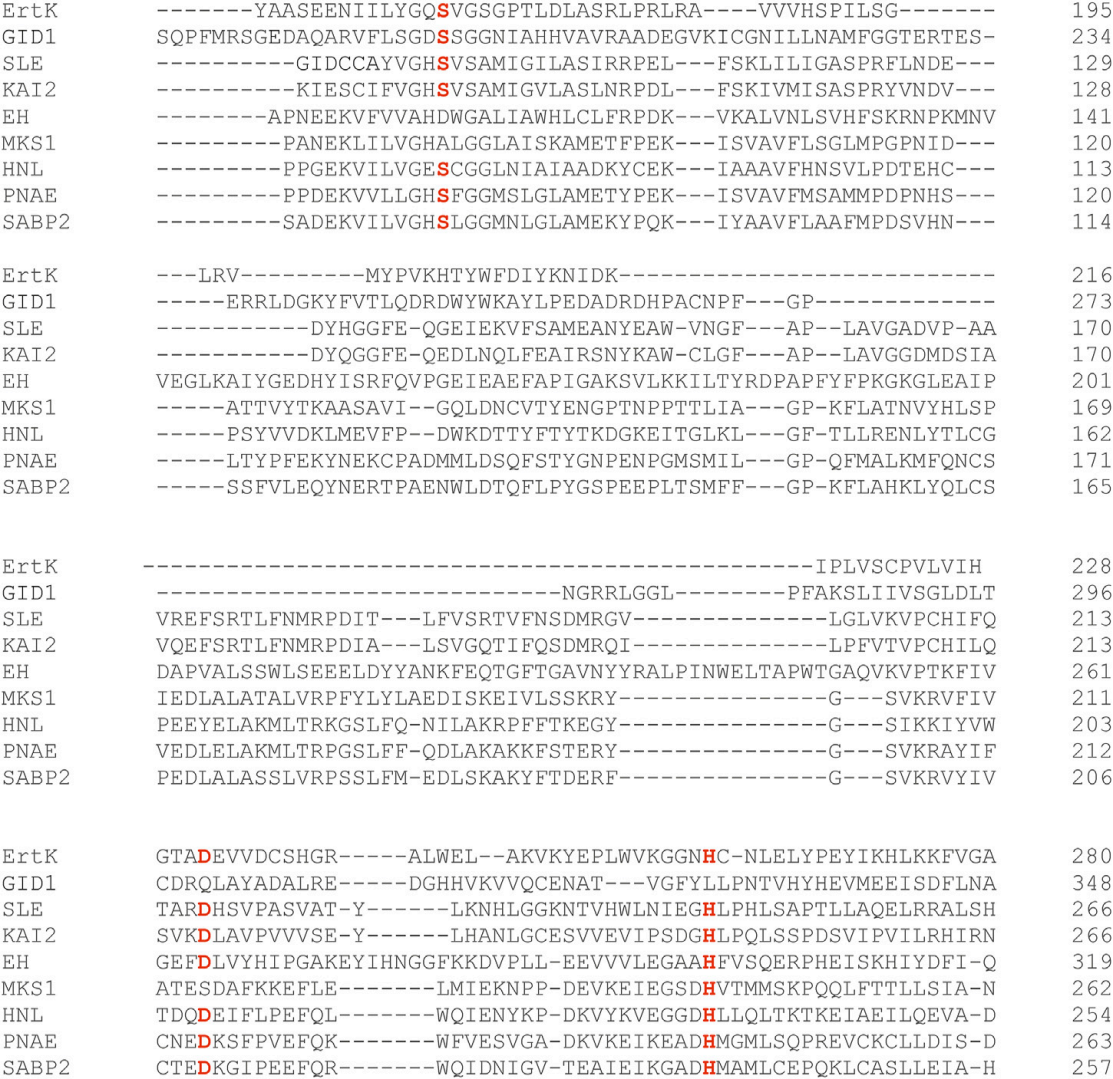
Alignment of the suggested ErtK polypeptide with a number of alpha/beta hydrolase plant proteins with known structure (Mindrebo et al., 2016). SABP2 - Salicylic acid binding protein (NP_001312442.1). HNL - Hydroxynitrile lyase (XP_021647581.1). MKS1- Methyl ketone synthase 1 (NP_001333340.1). PNAE - Polyneuridine aldehyde esterase (AAF22288.1). EH- Epoxide hydrolase (NP_001275417.1). SLE- Probable strigolactone esterase DAD2 (AFR68698.1/J9U5U9). GID1—Gibberellin receptor GID1 (XP_040380112.1). KAI2 - Karrikin receptor (OAO98902).

Alpha/beta hydrolases are characterized by an active site composed of a catalytic triad of three involved amino-acid residues. Typically, a serine residue plays the role of a nucleophile in the reaction. The serine is activated by a catalytic base and a catalytic acid, which are typically a histidine and aspartate residue, respectively. To analyze if the ErtK candidate has a catalytic triad and to identify the involved residues, we performed an alignment of the ErtK candidate with several alpha/beta hydrolases with published protein structures (Mindrebo et al., 2016) (Figure 7). The alignment suggested that Ser-167, His-261 and D-232 are the essential amino-acid residues for the activity of the suggested ErtK hydrolase (Figure 7). The 4-bp deletion in *ert-k*.32 and *ert-k*.76 is located before the codon corresponding to His-261 and D-232, whereas the 1-bp insertion in *ert-k*.309 is located after.

## Discussion

Genetic validation of genes and genomic regions associated with target traits for crop improvement is possible today thanks to recent advancements in genomic studies of cereals and other crop plants. The game changer in barley research came in 2017 when the barley reference genome was published (Mascher et al., 2017). The aim of the current study was to identify and validate the genetic cause of the *ert-k.32* mutation present in the cultivar Pallas, which was the first induced mutant to be released as a commercial barley cultivar on the market (in 1958). Pallas was known for its superior stem stability and increased lodging resistance. The reason to include semi-dwarf traits in plant breeding is to protect the plants against lodging under high nitrogenous fertilizer regimes. However, mutations providing a shorter and more sturdy plant architecture often have pleotropic effects, which affects other traits such as heading day (Kuczyńska et al., 2013) and malt quality (Hellewell et al., 2000). Therefore, mutations giving a mild short-culm phenotype have been favored by plant breeders over mutations giving a stronger phenotype (Vu et al., 2010; Dockter and Hansson, 2015). In certain barley types, breeders introgressed loss-of-function alleles of the semi-dwarfing gene *Sdw1* (Dockter and Hansson, 2015; Xu et al., 2017). Other alleles, such as *uzu1*.a and *ari-e*.GP, are less widespread. The *uzu1*. a allele is a mutation in the brassinosteroid receptor and selected in East Asian cultivars (Chono et al., 2003; Dockter et al., 2014). The Scottish malting cultivar Golden Promise contains the *ari-e*.GP mutation, which is a one-bp insertion in the gene encoding the alpha-subunit of a heterotrimeric G protein, and the semi-dwarfing allele of *HvAPETALA2* called *Zeo2* (Houston et al., 2013; Braumann et al., 2018). In comparison, mutations in *Ert-k* are relatively mild and we therefore believe that *ert-k* mutant alleles would be good alternatives to *sdw1* and other alleles to provide lodging resistance also in modern barley lines. Since the *ert-k* mutant phenotype is less obvious to recognize without training, the genetic validation of the *Ert-k* gene and the *ert-k.32* allele is important to provide genetic markers that can be followed in breeding programs rather than tracking ert-k mutant alleles by phenotyping. We validated that the 4-bp deletion in HORVU.MOREX.r3.6HG0574880 of *ert-k*.32 was not present in any analyzed Bonus cultivar or any cultivar included in the pedigree of Bonus. Therefore, we suggest the 4-bp deletion to be the genetic deficiency responsible for the stem stability and increased lodging resistance of *ert-k*.32, Pallas, Hellas, Senat and Visir, which are carrying the mutation.

The suggested *Ert-k* gene (HORVU.MOREX.r3.6HG0574880) encodes a protein of the alpha/beta-hydrolase superfamily. Alpha/beta-hydrolases have a broad range of functions. For example, they play important roles in primary and secondary metabolism as peptidases, lipases, peroxidases, esterases, thioesterases and dehalogenases (Ollis et al., 1992; Nardini and Dijkstra, 1999; Mindrebo et al., 2016; Dimitriou et al., 2017). Although the primary structure of alpha/beta-hydrolases differ significantly, the superfamily possesses a very conserved three-dimensional core structure. The core fold of alpha/beta-hydrolases is made by a beta-sheet, which consists of eight beta-strands and is surrounded by alpha-helices. The acid-base-nucleophile catalytic triad of alpha/beta hydrolases represent the most conserved elements of the alpha/beta hydrolase fold (Ollis et al., 1992; Mindrebo et al., 2016; Denesyuk et al., 2020). We identified Ser-167, His-261 and Asp-232 as the likely amino-acid residues to be involved in the catalytic triad. Interestingly, the fold of alpha/beta-hydrolases is also found in the major structure of phytohormone and ligand receptors of the gibberellin, karrikin and strigolactone signaling pathways in plants (Hotelier et al., 2004; Ueguchi-Tanaka et al., 2005; Ueguchi-Tanaka et al., 2007; Hamiaux et al., 2012; Waters et al., 2015). The receptors do not have the catalytic activity of a classic alpha/beta hydrolase. It was reported that loss-of-function mutations in the gibberellin receptor encoded by GID1, which is known to have the fold of an alpha/beta-hydrolase, produces a dwarf phenotype (Ueguchi-Tanaka et al., 2005). The gibberellin receptor lacks the histidine residue of the canonical catalytic triad and therefore does not have catalytic activity (Mindrebo et al., 2016). The finding of a complete catalytic triad in ErtK suggests that we have identified an enzymatically active alpha/beta hydrolase, which affects plant architecture. A search for proteins similar to ErtK in The Arabidopsis Information Resource (TAIR) database revealed AT3G01690 as the most similar protein (62% identical amino-acid residues). This protein is an Alpha/Beta Hydrolase Domain-containing Protein 17-like acyl protein thioesterase (ABAPT), which functions as a de-S-acylation enzyme (Liu et al., 2021). A cyclic S-acylation and de-S-acylation is an important post-translational modification of proteins in eukaryotes. In the S-acylation process, a fatty acid such as palmitate is covalently attached to a cysteine residue in the target protein via a thioester bond (Zaballa and van der Goot, 2018). The modification controls the localization and function of the target protein in the cell under different conditions (Lanyon-Hogg et al., 2017). The S-acylation is a reversible biochemical process that is mediated oppositely by S-acyltransferases and de-S-acylation enzymes. We believe that we have identified a gene putatively encoding a de-S-acylation protein with thioesterase activity that has a mild regulatory effect on plant architecture that we suggest is of relevance for plant breeding. In order to further understand the function of the suggested *Ert-k* candidate gene product, the target protein needs to be identified. It is possible that such protein is associated with the signaling or metabolism of the classic plant hormones regulating plant architecture such as culm length or inflorescence density.

## Conclusion

In the present study, we have analyzed the genetic deficiency of Pallas (*ert-k.*32), which was the first induced barley mutant to be released on the market as a commercial cultivar. We suggest that the dwarf phenotype of Pallas is caused by a 4-bp deletion in the gene HORVU.MOREX.r3.6HG0574880, which encodes an alpha/beta-hydrolase. The identified gene can be used in marker assistant breeding for cultivars with improved lodging resistance based on the identified *Ert-k* candidate gene.

## Data Availability

The sequences reported in this paper have been deposited in the GenBank database [accession nos. OQ872378 (Bonus *Ert-k*), OQ872379 (Foma *Ert-k*), OQ872380(*ert-k*.32 *Ert-k*), OQ872381 (*ert-k*.76 *Ert-k*), and OQ872382 (*ert-k*.309 *Ert-k*)]. The WGS data used in this study can be found in ENA database (accession- PRJEB61628, secondary accession -ERP146712).

## References

[B1] Badr A. Müller K. Schäfer-Pregl R. El Rabey H. Effgen S. Ibrahim H. H. (2000). On the origin and domestication history of barley (*Hordeum vulgare*). Mol. Biol. Evol. 17, 499–510. 10.1093/oxfordjournals.molbev.a026330 10742042

[B2] Braumann I. Dockter C. Beier S. Himmelbach A. Lok F. Lundqvist U. (2018). Mutations in the gene of the Gα subunit of the heterotrimeric G protein are the cause for the *brachytic1* semi-dwarf phenotype in barley and applicable for practical breeding. Hereditas 155, 10. 10.1186/s41065-017-0045-1 28878591 PMC5583965

[B3] Chono M. Honda I. Zeniya H. Yoneyama K. Saisho D. Takeda K. (2003). A semidwarf phenotype of barley uzu results from a nucleotide substitution in the gene encoding a putative brassinosteroid receptor. Plant Physiol. 133, 1209–1219. 10.1104/pp.103.026195 14551335 PMC281616

[B4] Close T. J. Bhat P. R. Lonardi S. Wu Y. Rostoks N. Ramsay L. (2009). Development and implementation of high-throughput SNP genotyping in barley. BMC Genomics 4 (10), 582. 10.1186/1471-2164-10-582 PMC2797026 19961604

[B5] Denesyuk A. Dimitriou P. S. Johnson M. S. Nakayama T. Denessiouk K. (2020). The acid-base-nucleophile catalytic triad in ABH-fold enzymes is coordinated by a set of structural elements. PLoS One 15, e0229376. 10.1371/journal.pone.0229376 32084230 PMC7034887

[B6] Dimitriou P. S. Denesyuk A. Takahashi S. Yamashita S. Johnson M. S. Nakayama T. (2017). Alpha/beta-hydrolases: A unique structural motif coordinates catalytic acid residue in 40 protein fold families. Proteins 85, 1845–1855. 10.1002/prot.25338 28643343

[B7] Dockter C. Hansson M. (2015). Improving barley culm robustness for secured crop yield in a changing climate. J. Exp. Bot. 66, 3499–3509. 10.1093/jxb/eru521 25614659

[B8] Dockter C. Gruszka D. Braumann I. Druka A. Druka I. Franckowiak J. (2014). Induced variations in brassinosteroid genes define barley height and sturdiness, and expand the green revolution genetic toolkit. Plant Physiol. 166, 1912–1927. 10.1104/pp.114.250738 25332507 PMC4256852

[B9] Doyle J. (1991). “DNA protocols for plants taxonomy,” in Molecular techniques. Editors Hewitt G. M. Johnston A. W. B. Young J. P. W. (Berlin, Heidelberg: Springer), 283–293. 10.1007/978-3-642-83962-7_18

[B10] Druka A. Franckowiak J. Lundqvist U. Bonar N. Alexander J. Houston K. (2011). Genetic dissection of barley morphology and development. Plant Physiol. 155, 617–627. 10.1104/pp.110.166249 21088227 PMC3032454

[B11] Franckowiak J. D. Lundqvist U. (2012). Description of barley genetic stocks for 2012. Barley Genet. Newsl. 42, 36–792.

[B12] Gustafsson A. Hagberg A. Persson G. Wiklund K. (1971). Induced mutations and barley improvement. Theor. Appl. Genet. 41, 239–248. 10.1007/BF00277792 24430352

[B13] Gustafsson A. (1940). “The mutation system of the chlorophyll apparatus,” in Kgl Fysiografiska sällsk handl (Lund), N F Bd 51, Nr 11.

[B14] Hamiaux C. Drummond R. S. Janssen B J. Ledger S. E. Cooney J. M. Newcomb R. D. (2012). DAD2 is an α/β hydrolase likely to be involved in the perception of the plant branching hormone, strigolactone. Curr. Biol. 22, 2032–2036. 10.1016/j.cub.2012.08.007 22959345

[B15] Hellewell K. B. Rasmusson D. C. Gallo-Meagher M. (2000). Enhancing yield of semidwarf barley. Crop Sci. 40, 352–358. 10.2135/cropsci2000.402352x

[B16] Hotelier T. Renault L. Cousin X. Negre V. Marchot P. Chatonnet A. (2004). ESTHER, the database of the α/β -hydrolase fold superfamily of proteins. Nucleic Acids Res. 41, 32D145–32D147. 10.1093/nar/gkh141 PMC308875 14681380

[B17] Houston K. McKim S. M. Comadran J. Bonar N. Druka I. Uzrek N. (2013). Variation in the interaction between alleles of *HvAPETALA2* and microRNA172 determines the density of grains on the barley inflorescence. *Proc. Natl. Acad. Sci*. U. S. A. 110, 16675–16680. 10.1073/pnas.1311681110 24065816 PMC3799380

[B18] International Barley Genome Sequencing Consortium. Mayer K. F. Waugh R. Brown J. W. Schulman A. Langridge P. (2012). A physical, genetic and functional sequence assembly of the barley genome. Nature. 491, 711–716. 10.1038/nature11543 23075845

[B19] Kuczyńska A. Surma M. Adamski T. Mikołajczak K. Krystkowiak K. Ogrodowicz P. (2013). Effects of the semi-dwarfing *sdw1/denso* gene in barley. J. Appl. Genet. 54, 381–390. 10.1007/s13353-013-0165-x 23975516 PMC3825292

[B20] Lanyon-Hogg T. Faronato M. Serwa R. A. Tate E. W. (2017). Dynamic protein acylation: New substrates, mechanisms, and drug targets. Trends Biochem. Sci. 42, 566–581. 10.1016/j.tibs.2017.04.004 28602500

[B21] Li H. Durbin R. (2010). Fast and accurate long-read alignment with Burrows-Wheeler transform. Bioinformatics 26, 589–595. 10.1093/bioinformatics/btp698 20080505 PMC2828108

[B22] Li H. Handsaker B. Wysoker A. Fennell T. Ruan J. Homer N. (2009). The sequence alignment/map format and SAMtools. Bioinformatics 25, 2078–2079. 10.1093/bioinformatics/btp352 19505943 PMC2723002

[B23] Li H. (2011). A statistical framework for SNP calling, mutation discovery, association mapping and population genetical parameter estimation from sequencing data. Bioinformatics 27, 2987–2993. 10.1093/bioinformatics/btr509 21903627 PMC3198575

[B24] Liu X. Li M. Li Y. Chen Z. Zhuge C. Ouyang Y. (2021). An ABHD17-like hydrolase screening system to identify de-S-acylation enzymes of protein substrates in plant cells. Plant Cell. 33, 3235–3249. 10.1093/plcell/koab199 34338800 PMC8505870

[B25] Lundqvist U. (1992). Mutation research in barley. Dissertation. Alnarp, Sweden: Swedish University of Agricultural Sciences.

[B26] Mascher M. Schuenemann V. J. Davidovich U. Marom N. Himmelbach A. Hübner S. (2016). Genomic analysis of 6,000-year-old cultivated grain illuminates the domestication history of barley. Nat. Genet. 48, 1089–1093. 10.1038/ng.3611 27428749

[B27] Mascher M. Gundlach H. Himmelbach A. Beier S. Twardziok S. O. Wicker T. (2017). A chromosome conformation capture ordered sequence of the barley genome. Nature 544 (7651), 427–433. 10.1038/nature22043 28447635

[B28] Mascher M. Wicker T. Jenkins J. Plott C. Lux T. Koh C. S. (2021). Long-read sequence assembly: A technical evaluation in barley. Plant Cell. 33, 1888–1906. 10.1093/plcell/koab077 33710295 PMC8290290

[B29] Matyszczak I. Tominska M. Zakhrabekova S. Dockter C. Hansson M. (2020). Analysis of early-flowering genes at barley chromosome 2H expands the repertoire of mutant alleles at the *Mat-c* locus. Plant Cell. Rep. 39, 47–61. 10.1007/s00299-019-02472-4 31541262 PMC6960220

[B30] McLaren W. Gil L. Hunt S. E. Riat H. S. Ritchie G. R. Thormann A. (2016). The Ensembl variant effect predictor. Genome Biol. 17, 122. 10.1186/s13059-016-0974-4 27268795 PMC4893825

[B31] Mindrebo J. T. Nartey C. M. Seto Y. Burkart M. D. Noel J. P. (2016). Unveiling the functional diversity of the alpha/beta hydrolase superfamily in the plant kingdom. Curr. Opin. Struct. Biol. 41, 233–246. 10.1016/j.sbi.2016.08.005 27662376 PMC5687975

[B32] Muller H. J. (1927). Artificial transmutation of the gene. Science 66 (1699), 84–87. 10.1126/science.66.1699.84 17802387

[B33] Muller H. J. (1928). “The problem of genetic modification,” in Verh. V. Int. Kongr. Vererbungswiss. Zeitschrift für induktive Abstammungs-und Vererbungslehre Suppl. I, 234–260.

[B34] Nardini M. Dijkstra B. W. (1999). α/β hydrolase fold enzymes: The family keeps growing. Curr. Opin. Struct. Biol. 9, 732–737. 10.1016/s0959-440x(99)00037-8 10607665

[B35] Ollis D. L. Cheah E. Cygler M. Dijkstra B. Frolow F. Franken S. M. (1992). The alpha/beta hydrolase fold. Protein Eng. 5, 197–211. 10.1093/protein/5.3.197 1409539

[B36] Pennacchio L. A. Bickmore W. Dean A. Nobrega M. A. Bejerano G. (2013). Enhancers: Five essential questions. Nat. Rev. Genet. 14, 288–295. 10.1038/nrg3458 23503198 PMC4445073

[B37] Skov Kristensen P. Dockter C. Lundqvist U. Lu Q. Gregersen P. L. Thordal-Christensen H. (2016). Genetic mapping of the barley lodging resistance locus *Erectoides-k* . Plant Breed. 135, 420–428. 10.1111/pbr.12377

[B38] Stuart D. Sandström M. Youssef H. M. Zakhrabekova S. Jensen P. E. Bollivar D. (2021). Barley *Viridis-k* links an evolutionarily conserved C-type ferredoxin to chlorophyll biosynthesis. Plant Cell. 33, 2834–2849. 10.1093/plcell/koab150 34051099 PMC8408499

[B39] Ueguchi-Tanaka M. Ashikari M. Nakajima M. Itoh H. Katoh E. Kobayashi M. (2005). *Gibberellin Insensitive DWARF1* encodes a soluble receptor for gibberellin. Nature 437 (7059), 693–698. 10.1038/nature04028 16193045

[B40] Ueguchi-Tanaka M. Nakajima M. Katoh E. Ohmiya H. Asano K. Saji S. (2007). Molecular interactions of a soluble gibberellin receptor, GID1, with a rice DELLA protein, SLR1, and gibberellin. Plant Cell. 19, 2140–2155. 10.1105/tpc.106.043729 17644730 PMC1955699

[B41] Vu G. T. Wicker T. Buchmann J. P. Chandler P. M. Matsumoto T. Graner A. (2010). Fine mapping and syntenic integration of the semi-dwarfing gene *sdw3* of barley. Funct. Integr. Genomics, 10509–10521. 10.1007/s10142-010-0173-4 20464438

[B42] Waters M. T. Scaffidi A. Moulin S. L. Sun Y. K. Flematti G. R. Smith S. M. (2015). A *Selaginella moellendorffii* ortholog of karrikin insensitive2 functions in arabidopsisin arabidopsis development but cannot mediate responses to karrikins or strigolactones. Plant Cell. 27, 1925–1944. 10.1105/tpc.15.00146 26175507 PMC4531350

[B43] Xu Y. Jia Q. Zhou G. Zhang X. Q. Angessa T. Broughton S. (2017). Characterization of the *sdw1* semi-dwarf gene in barley. BMC Plant Biol. 17 (1), 11. 10.1186/s12870-016-0964-4 28086794 PMC5237212

[B44] Zaballa M. E. van der Goot F. G. (2018). The molecular era of protein S-acylation: Spotlight on structure, mechanisms, and dynamics. Crit. Rev. Biochem. Mol. Biol. 53, 420–451. 10.1080/10409238.2018 29999430

[B45] Zakhrabekova S. Gough S. P. Braumann I. Müller A. H. Lundqvist J. Ahmann K. (2012). Induced mutations in circadian clock regulator *Mat-a* facilitated short-season adaptation and range extension in cultivated barley. *Proc. Natl. Acad. Sci*. U. S. A. 109:4326–4331. 10.1073/pnas.1113009109 22371569 PMC3306670

[B46] Zakhrabekova S. Dockter C. Ahmann K. Braumann I. Gough S. P. Wendt T. (2015). Genetic linkage facilitates cloning of *Ert-m* regulating plant architecture in barley and identified a strong candidate of *Ant1* involved in anthocyanin biosynthesis. Plant Mol. Biol. 88, 609–626. 10.1007/s11103-015-0350-x 26228300

