# Supplementary material for: Identification of a candidate dwarfing gene in Pallas, the first commercial barley cultivar generated through mutational breeding
Source: Front Genet. 2023 Jul 4;14:1213815. doi: 10.3389/fgene.2023.1213815 (PMC10352844; doi:10.3389/fgene.2023.1213815)
Supplement: Supplementary file 1 [file DataSheet1.PDF]

Supplemental Table 1. Available *ert* mutants at the Nordic Genetic Resource Center (www.nordgen.org) at the 31 identified *ert* loci. The table shows number of available original *ert* alleles, identified function when known and alternative locus names.

Locus	Number of alleles	Identified function	Alternative locus name
ert-a	43		
ert-b	10		
ert-c	40		
ert-d	27		
ert-e	6		
ert-f	3		
ert-g	10		
ert-h	2		
ert-i	3		
ert-ii	1	Brassinosteroid-receptor - HvBRI1 (Dockter et al., 2014)	*uzu1*
ert-j	3		
ert-k	8	Alpha/beta-hydrolases superfamily protein (this study)	
ert-l	11		
ert-m	20	Leucine-rich repeat receptor-like kinase - HvERECTA (Zakhrabekova et al., 2015)	
ert-n	9		
ert-o	1	Circadian clock regulator - HvELF3 (Zakhrabekova et al., 2012)	*mat-a, eam1*
ert-p	6		
ert-q	3		
Ert-r	4	APETALA2 (AP2)-like transcription factor - HvAP2 (Houston et al., 2013)	*Zeo1, Zeo2, Zeo3*
ert-s	1		
ert-t	2	Brassinosteroid-6-oxidase - HvBRD (Dockter et al., 2014)	*ari-u, brh3*
ert-u	1	Δ 5-sterol-Δ 24-reductase -	*ari-o, brh14, brh16, ert-zd*

		Diminuto HvDIM (Dockter et al., 2014)	
ert-v	2		
ert-x	1		
ert-y	1		
ert-z	1		
ert-za	2		
ert-zb	1		
ert-zc	1		
ert-zd	1	Δ 5-sterol-Δ 24-reductase - Diminuto HvDIM (Dockter et al., 2014)	*ari-o, brh14, brh16, ert-u*
ert-ze	1		

Supplemental Table 2. Barley spring cultivars analyzed in this study.

Barley cultivar name	NGB number
Bonus	1489
Bonus	14682
Bonus	131146
Bonus	131182
Bonus	131685
Bonus	131708
Bonus	131725
Foma	1492
Gull	1480
Opal Abed	4619
Maja Abed	8815
Binder Abed	9440
Guld Svalöf	9454
Seger	9467
Hanna	9550
Hanna	19108
Pallas	1490
Pallas	4959
Pallas	8889
Pallas	14663
Jenny	1511
Hellas	1495
Senat	1503
Visir	1496

Supplemental Table 3. Origin of the eight available *ert-k* mutants. Five mutants, which had not been induced by neutrons, were selected for whole genome DNA sequencing.

Mutant	Mother cultivar	Year of isolation	Mutagen	NGB number
ert-k.32	Bonus	1947	X-rays	112633
ert-k.76	Bonus	1955	gamma-rays	112675
ert-k.93	Bonus	1956	neutrons	112692
ert-k.302	Foma	1959	neutrons	112819
ert-k.309	Foma	1959	X-rays	112825
ert-k.435	Foma	1960	ethyl methanesulfonate	112951
ert-k.459	Foma	1961	neutrons	112974
ert-k.477	Foma	1961	ethyl methanesulfonate	112993

Supplemental Table 4. Primers used for PCR amplifications of HORVU.MOREX.r3.6HG0574880, its intergenic region and *Ert-r*.

**HORVU.MOREX.r3.6HG0574880 genomic DNA**		
Primer name	Sequence (5' to 3')	Size of fragment
F1	CAACCCAGACCGGTAATCCC	939 bp
R1	CCAGGCACTAGCACACATGA	
F2	TCTGGGGATCAAAGCCACAC	811 bp
R2	AGCAGTTACGGGTGTTACGG	
F3	ACTGGAGGTGACCGAAGACT	907 bp
R3	GCACAAACATGGAGGCATGG	
F4	GGCCACTAGTCTTAGCGCAA	948 bp
R4	GGGTCCGACCACTATCTTCC	
F5	GCTGCCTCCCGTGCTAATTA	787 bp
R5	ACTGCTCTCAAACGAGGCAA	
F6	TGGGCAATCTTCTGGAAAGGT	843 bp
R6	CCGCCAGAAATACAGTGATGG	
F7	ACGGGCACACATTTGCATTC	993 bp
R7	TAGCTCGTGGTCTGTCAGGA	
F8	GGCATTGTGGGAACTCGCTA	768 bp
R8	AGGAACACAAGAAAACAGAAGCA	
**HORVU.MOREX.r3.6HG0574880 cDNA**		
cDNA-F1	AAAGGGAAGGAGGGCAGC	1006 bp
cDNA-R1	GCAACCTGACAGCCTTCATC	
**Intergenic region of HORVU.MOREX.r3.6HG0574880**		
intergenic 477-F1	TGCTCCTCAAGATAATGTCGTT	

intergenic 477-R1	GCTTGCATGGATCTGGTTGC	320 bp
**Genotyping of F_1 plants**		
wt-F1	TGTTGTACATAGTCCAATTTTGTCTG	400 bp
wt-R1	CCTGCTTGGTATTTTCTCCACT	
mut-F1	CCAATTTTGGCTTAAGAGTGATGT	720 bp
mut-R1	GGTAGTATACAAACTCAACTCATGGT	
Ert-r **(*APETALA2* (*AP2*)) - HORVU.MOREX.r3.2HG0204770**		
Ert-r-F1	CACCCTGCAACAAACTACTGC	909 bp
Ert-r-R1	TCGCTCTCACCCAATCAATCA	

Supplemental Table 5. Analyzed Pallas accessions and barley cultivars with Pallas in their pedigrees. Lines were ordered from Nordic Genetic Stock Center (www.nordgen.org).

Release year	Cultivar	NGB number	Pedigree
1958	Pallas	NGB1490	Mutation selected from X-ray treated Bonus
1958	Pallas	NGB4959	Mutation selected from X-ray treated Bonus
1958	Pallas	NGB8889	Mutation selected from X-ray treated Bonus
1958	Pallas	NGB14663	Mutation selected from X-ray treated Bonus
1980	Jenny	NGB1511	Kristina x (Hellas 2 x (Pallas 5 x Rupee)
1967	Hellas	NGB1495	Pallas x Herta
1974	Senat	NGB1503	(Triple Awn Lemma x Pallas) x Hellas
1970	Visir	NGB1496	Pallas x Long Glumes
